# Protocol for the Preparation of a Recombinant Treacle Fragment for Liquid–Liquid Phase Separation (LLPS) Assays

**DOI:** 10.21769/BioProtoc.5439

**Published:** 2025-09-20

**Authors:** Nadezhda V. Petrova, Konstantin I. Balagurov, Sergey V. Razin, Artem K. Velichko

**Affiliations:** 1 Institute of Gene Biology Russian Academy of Science, Moscow, Russia; 2 Biological Faculty, Lomonosov Moscow State University, Moscow, Russia

**Keywords:** Treacle, Liquid–liquid phase separation (LLPS), Biomolecular condensates, Nucleolus, Low-complexity repeat (LCR), Peptide purification, Intrinsically disordered protein, Bacterial expression, Maltose-binding protein (MBP) fusion protein, Tobacco Etch Virus (TEV) protease

## Abstract

Liquid–liquid phase separation (LLPS) underlies the spatial organization of the nucleolus, a membraneless organelle responsible for ribosomal RNA (rRNA) transcription and ribosome subunit assembly. One of the key proteins involved in the formation of the fibrillar center of the nucleolus is the treacle, an intrinsically disordered protein that contains low-complexity repeats enriched in charged amino acid residues. In this work, we present a detailed protocol for the bacterial expression and purification of a recombinant fragment of treacle comprising two tandem low-complexity repeat (LCR) modules, with a total length of 136 amino acids. This fragment is intended for subsequent in vitro investigation of its ability to undergo LLPS. The described method enables the production of a soluble, biochemically pure protein preparation suitable for studying the mechanisms of spontaneous condensate formation in a cell-free system. This approach allows for the controlled modeling and quantitative evaluation of the contribution of low-complexity sequences to the phase behavior of treacle, independently of its interactions with cellular partners in vivo.

Key features

• Protocol describes the expression and purification of a soluble treacle fragment containing two LCR motifs, suitable for in vitro LLPS studies.

• Avoids complications associated with full-length treacle expression by using a minimal, well-behaved construct.

• Produces biochemically pure protein compatible with phase separation assays under controlled buffer and crowding conditions.

• Applicable for dissecting sequence features driving electrostatically mediated LLPS in nucleolar IDR/LCR proteins.

## Graphical overview


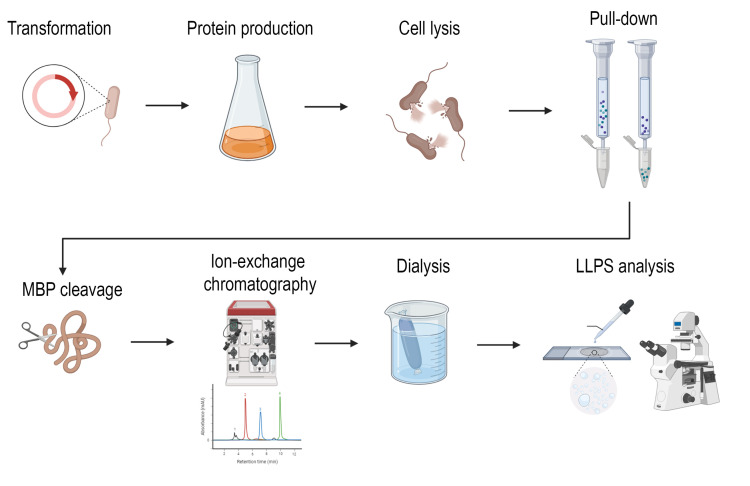


## Background

The nucleolus is a dynamically organized, membraneless organelle that facilitates the transcription of ribosomal DNA (rDNA) and the assembly of ribosomal subunits [1]. Its structural organization is driven by liquid–liquid phase separation (LLPS), which gives rise to three distinct subcompartments: the fibrillar center (FC), the dense fibrillar component (DFC), and the granular component (GC) [2,3]. LLPS is mediated by multivalent interactions among macromolecules such as RNA and proteins containing intrinsically disordered regions (IDRs) and low-complexity regions (LCRs) [4,5].

**Figure 1.**
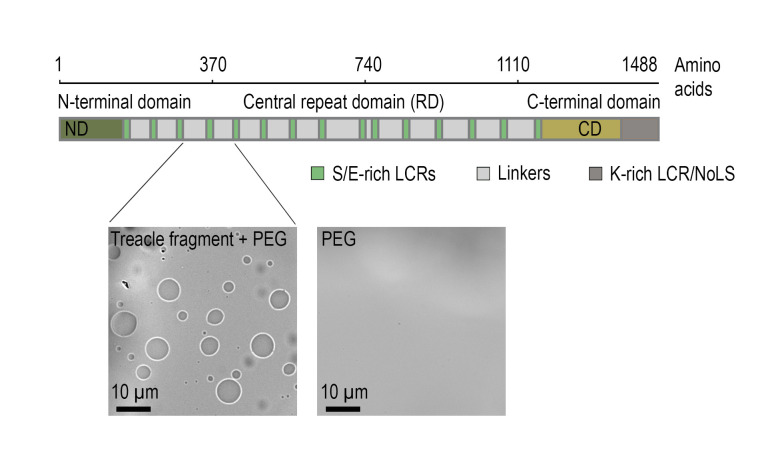
Treacle drives the formation of nuclear condensates. The top part of the panel shows the structure of the most common isoform of treacle. Treacle isoform d (1,488 amino acids, 152 kDa, NP_001128715.1) is encoded by the treacle transcript variant 4. It is an intrinsically disordered protein with N-terminal (ND, 1–83 aa) and C-terminal (CD, 1,121–1,488 aa) regions and a central repeated domain (RD, 83–1,121 aa) consisting of 15 low complexity regions (LCR) interspersed with disordered linker sequences. The bottom part of the panel shows that the purified recombinant fragment of treacle undergoes condensation in vitro in the presence of 5% polyethylene glycol (PEG).

The protein treacle (TCOF1) is a key component of the fibrillar center. It is an intrinsically disordered protein composed of 1,488 amino acids, with a central region containing 15 tandem LCR blocks enriched in charged residues (Figure 1) [6]. These repetitive modules alternate between positively and negatively charged segments, providing a molecular basis for LLPS via electrostatic interactions [7].

In our previous work, we demonstrated that a recombinant treacle fragment comprising 136 amino acids and encompassing two representative LCR repeats is capable of forming biomolecular condensates in vitro in aqueous solution (Figure 1). These findings confirmed that even a minimal segment of treacle is sufficient to drive LLPS outside the cellular context [7].

In the present study, we provide a detailed protocol for the expression and purification of this treacle fragment, from bacterial production to the isolation and preparation of a biochemically stable and functional protein. This method can be applied to systematically investigate the structural determinants of LLPS and serves as a model system for studying the molecular mechanisms of condensate assembly by LCR-containing proteins.

## Materials and reagents

**Biological materials**

1. *Escherichia coli* BL21(DE3) cells for protein expression (Novagen 69450-M)

*Note: Cell cultures were stored long-term in 15%–25% glycerol at -80 °C or short-term on LB agar plates at 4 °C*

2. pMAL_TCOF 2× LCR (obtained in the article [7]); store at -20 °C

*Note: A fragment of wt treacle (amino acids 291–426) was amplified by PCR and subcloned into the C-terminus of the pMAL vector, which encodes an N-terminal MBP followed by a TEV protease cleavage site. The plasmid pMAL_TCOF 2× LCR was generated in our laboratory and is available upon request.*

DNA sequence of TCOF 2LCR:

5’-GTAAAGGCCTCTGAAAAAATTCTCCAGGTCAGAGCTGCCTCAGCCCCTGCCAAGGGGACCCCTGGGAAAGGGGCTACCCCAGCACCCCCTGGGAAGGCAGGGGCTGTAGCCTCCCAGACCAAGGCAGGGAAGCCAGAGGAGGACTCAGAGAGCAGCAGCGAGGAGTCATCTGACAGTGAGGAGGAGACGCCAGCTGCCAAGGCCCTGCTTCAGGCGAAGGCCTCAGGAAAAACCTCTCAGGTCGGAGCTGCCTCAGCCCCTGCCAAGGAGTCCCCCAGGAAAGGAGCTGCCCCAGCGCCCCCTGGGAAGACAGGGCCTGCAGTTGCCAAGGCCCAGGCGGGGAAGCGGGAGGAGGACTCGCAGAGCAGCAGCGAGGAATCGGACAGTGAGGAGGAGGCGCCTGCTCAG

encodes 136 amino acids (aa):

VKASEKILQVRAASAPAKGTPGKGATPAPPGKAGAVASQTKAGKPEEDSESSSEESSDSEEETPAAKALLQAKASGKTSQVGAASAPAKESPRKGAAPAPPGKTGPAVAKAQAGKREEDSQSSSEESDSEEEAPAQ

**Reagents**

1. Absolute ethanol; store at room temperature (RT) in a flammable liquid storage cupboard

**Caution**: This chemical is flammable.

2. Acrylamide (PanReac AppliChem, catalog number: A1090); store at 4 °C

**Caution**: This chemical is toxic.

3. Ampicillin (PanReac AppliChem, catalog number: A0839); store at 4 °C

**Caution**: Ampicillin is a sensitiser.

4. Amylose resin (New England BioLabs, catalog number: E8021S); store at 4 °C

5. Bis-acrylamide (PanReac AppliChem, catalog number: A3636); store at 4 °C

**Caution**: This chemical is toxic.

6. Bromophenol blue (Sigma-Aldrich, Merck, catalog number: B-8026); store at RT

7. Calcium chloride (CaCl_2_) dihydrate (Sigma-Aldrich, Merck, catalog number: 10035-04-8); store at RT

8. dH_2_O

9. D(+)-Maltose monohydrate (neoFroxx, catalog number: 1495GR); store at 4 °C

10. Dithiothreitol (DTT) (Sigma-Aldrich, Merck, catalog number: D9779); store at 4 °C

**Caution**: DTT can cause skin and eye irritation, and is harmful if swallowed or inhaled

11. Glucose (Amresco, catalog number: Am-O188); store at 4 °C

12. Glycerol (PanReac AppliChem, catalog number: H-0403); store at RT

13. Glycine (Sigma-Aldrich, Merck, catalog number: 50046); store at RT

14. Hydrochloric acid (HCl) 37% (PanReac AppliChem, catalog number: 131020); store at RT

**Caution**: This chemical causes burns.

15. Isopropyl thiogalactose (IPTG) (neoFroxx, catalog number: 1122GR); store at -20 °C

16. Magnesium chloride hexahydrate (Sigma-Aldrich, Merck, catalog number: 63068); store at RT

17. Magnesium sulfate (MgSO_4_) anhydrous (Sigma-Aldrich, Merck, catalog number: M7506); store at RT

18. Manganese(II) chloride (MnCl_2_) tetrahydrate (Sigma-Aldrich, Merck, catalog number: 13446-34-9); store at RT

19. Metanol (Merck, catalog number: 1060182500); store at RT in a flammable liquid storage cupboard

**Caution**: This chemical is flammable.

20. Nonidet P40 (NP40) (Thermo Scientific, catalog number: 85124); store at RT

21. One-Step Blue Protein Gel Stain (Biotium, catalog number: 21003); store at 4 °C

22. PageRuler prestained protein ladder (Thermo Scientific, catalog number: 26616); store at -20 °C

23. Polyethylene glycol 8000 (PEG 8000) (Sigma-Aldrich, Merck, catalog number: 5413); store at RT

24. Phosphatase inhibitor cocktail (100×) (Selleckchem, catalog number: B15001-A, B15001-B); store at -20 °C

25. PIPES (Sigma-Aldrich, Merck, catalog number: 13446-34-9); store at RT

26. Phenylmethylsulfonyl fluoride (PMSF) (Amresco, catalog number: Am-0754); store at -20 °C

27. Potassium phosphate dibasic (Sigma-Aldrich, Merck, catalog number: 60353); store at RT

28. Potassium chloride (KCl) (Sigma-Aldrich, Merck, catalog number: 7447-40-7); store at RT

29. Potassium phosphate monobasic (Sigma-Aldrich, Merck, catalog number: P5655); store at RT

30. Protease inhibitor cocktail (100**×**) (Selleckchem, catalog number: B14001); store at -20 °C

31. Ammonium persulfate (APS) (Sigma-Aldrich, Merck, catalog number: 09913); store at RT

32. Qubit^TM^ Protein BR Assay kit (Invitrogen, catalog number: A50668); store at RT

33. Sodium dodecyl sulfate (SDS) (Sigma-Aldrich, Merck, catalog numbers: L4390, L3771); store at RT

**Caution**: This can irritate skin and eyes and is harmful to aquatic life.

34. Sodium chloride (NaCl) (Sigma-Aldrich, Merck, catalog number: S3014); store at RT

35. Sodium hydroxide (NaOH) (Sigma-Aldrich, Merck, catalog number: S8045); store at RT

**Caution**: This chemical causes burns.

36. N,N,N',N'-tetramethylethylenediamine (TEMED) (Sigma-Aldrich, Merck, catalog number: T9281); store at 4 °C **Caution**: This chemical has a strong odor and is flammable and toxic.

37. Tobacco Etch Virus (TEV) protease (New England BioLabs, catalog number: P8112S); store at -20 °C

38. Triptone (Amresco, catalog number: Am-J859-1.0); store at RT

39. Tris base (Sigma-Aldrich, Merck, catalog number: 10708976001); store at RT

40. Trisodium citrate dihydrate (Na_3_C_6_H_5_O_7_) (Fisher, catalog number: 6132-04-3); store at RT

41. Yeast extract (Biospringer, catalog number: H-0601MG); store at RT

42. β-mercaptoethanol (Sigma-Aldrich, Merck, catalog number: M3148); store at 4 °C

**Caution**: This chemical has a strong odor and is toxic.

43. 1,6-hexandiol (Sigma-Aldrich, Merck, catalog number: 240117), store at RT

**Solutions**

1. 1.5% (w/v) agar LB plates with ampicillin and glucose (see Recipes)

2. 1.5% (w/v) agar LB plates without antibiotic (see Recipes)

3. 10× Phosphate buffer (see Recipes)

4. 1× Phosphate buffer (see Recipes)

5. Terrific Broth medium (see Recipes)

6. TB buffer (see Recipes)

7. Start buffer (see Recipes)

8. Wash buffer (see Recipes)

9. Elution buffer with maltose (see Recipes)

10. Maltose-free elution buffer (see Recipes)

11. Buffer A (see Recipes)

12. Buffer B (see Recipes)

13. PIPES 250 mM, pH 7.5 (see Recipes)

14. Tris-HCl, 1 M, pH 7.5 (see Recipes)

15. Tris-HCl, 1 M, pH 7.4 (see Recipes)

16. Tris-HCl, 1 M, pH 6.8 (see Recipes)

17. NaCl, 4 M (see Recipes)

18. NaOH, 5 M (see Recipes)

19. CaCl_2_, 1.5 M (see Recipes)

20. MnCl_2_, 1 M (see Recipes)

21. KCl, 2.5 M (see Recipes)

22. MgSO_4_, 1 M (see Recipes)

23. Maltose, 1 M (see Recipes)

24. Na_3_C_6_H_5_O_7_, 1 M (see Recipes)

25. IPTG, 1 M (see Recipes)

26. DTT, 1 M (see Recipes)

27. Ampicillin 1,000× (see Recipes)

28. Glucose, 40% (w/v) (see Recipes)

29. SDS, 10% (w/v) (see Recipes)

30. APS, 10% (w/v) (see Recipes)

31. 4× Loading buffer (SLB) (see Recipes)

32. 1× Electrode buffer (Tris-Glycine/SDS) pH 8.3 (see Recipes)

33. Acrylamide/Bis-acrylamide, 30% solution (see Recipes)

34. Resolving gel buffer pH 8.8 (see Recipes)

35. Stacking gel buffer pH 6.8 (see Recipes)

36. 20% PEG 8000 (see Recipes)

**Recipes**

**1. 1.5% (w/v) agar LB plates with ampicillin and glucose**

**Table:** 

Reagent	Final concentration	Quantity or Volume
Triptone		2 g
Yeast extract		1 g
NaCl		2 g
Agar		3 g
Glucose (40%)	0.5% (w/v)	2.5 mL
Adjust to pH 7.0 with NaOH 5 M		67 μL
dH_2_O	to 200 mL	
Ampicillin (1,000×)	100 μg/mL	2 mL
Total		**200 mL**

Autoclave at 121 °C for 20 min. Refrigerate to 50 °C before adding ampicillin to a final concentration of 100 μg/mL and glucose to a final concentration of 0.5%. Pour 25–35 mL of medium into 85 mm petri dishes. Let the agar harden. Store at 4 °C for up to 1 month or at room temperature for up to 1 week. Glucose is needed to suppress background transcription from the promoter.

**2. 1.5% (w/v) agar LB plates without antibiotic**

**Table:** 

Reagent	Final concentration	Quantity or Volume
Triptone		2 g
Yeast extract		1 g
NaCl		2 g
Agar		3g
Adjust to pH 7.0 with NaOH 5 M		67 μL
dH_2_O	to 200 mL	
Total		**200 mL**

Autoclave at 121 °C for 20 min. Refrigerate to 50 °C. Pour 25–35 mL of medium into 85 mm petri dishes. Let the agar harden. Store at 4 °C for two weeks.

**3. 10× Phosphate buffer**

**Table:** 

Reagent	Final concentration	Quantity or Volume
K_2_HPO_4_·3H_2_O	720 mM	125 g
KH_2_PO_4_	170 mM	23 g
MgSO_4 _(1 M)	5 mM	5 mL
dH_2_O	n/a	90 mL
Total		**1 L**

Autoclave at 121 °C for 20 min. Sterilize by filtration 1 M MgSO_4_ and add directly to the solution before use.

**4. 1× Phosphate buffer**

**Table:** 

Reagent	Final concentration	Quantity or Volume
10× Phosphate buffer	1/10	100 mL
dH_2_O	n/a	900 mL
Total		**1 L**

**5. Terrific Broth medium**

**Table:** 

Reagent	Final concentration	Quantity or Volume
Triptone		12 g
Yeast extract		24 g
Glycerol	0.5% (w/v)	5 mL
dH_2_O	n/a	995 mL
Total		**1 L**

Autoclave at 121 °C for 15 min. Store at RT.

**6. TB buffer**

**Table:** 

Reagent	Final concentration	Quantity or Volume
PIPES pH 7.5 (250 mM)	10 mM	2 mL
CaCl_2_ (1.5 M)	15 mM	0.5 mL
KCl (2.5 M)	250 mM	5 mL
MnCl_2_ (1 M)	55 mM	2.75 mL
dH_2_O	n/a	39.75 mL
Total		**50 mL**

Filter sterilize using a 0.22 μm filter. Store at 4 °C.

**7. Start buffer**

**Table:** 

Reagent	Final concentration	Quantity or Volume
Tris-HCl pH 7.5 (1 M)	20 mM	4 mL
NaCl (4 M)	150 mM	7.5 mL
MgCl_2_ (1 M)	10 mM	2 mL
NP40 (20%)	0.1% (v/v)	1 mL
Glycerol	10%	20 mL
dH_2_O	n/a	161.5 mL
Total		**200 mL**
**Add before use**		
DTT (1 M)	1 mM	200 μL
Protease inhibitor cocktail (100**×**)	1**×**	2 mL
Phosphatase inhibitor cocktail (100**×**)	1**×**	2 mL

Prepare fresh. The prepared solution can be stored at 4 °C and used for 6 months.

DTT (to a final concentration of 1 mM), protease inhibitor cocktail (to a final concentration of 1**×**), and phosphatase protease inhibitor cocktail (to a final concentration of 1**×**) are added to the start buffer strictly before use.

**8. Wash buffer**

**Table:** 

Reagent	Final concentration	Quantity or Volume
Tris-HCl pH 7.5 (1 M)	20 mM	4 mL
NaCl (4 M)	500 mM	25 mL
MgCl_2 _(1 M)	10 mM	2 mL
NP40 (20%)	0.1% (v/v)	1 mL
Glycerol	10%	20 mL
dH_2_O	n/a	148 mL
Total		**200 mL**
**Add before use**		
DTT (1 M)	1 mM	200 μL

Prepare fresh. The prepared solution can be stored at 4 °C and used for 6 months. DTT (to a final concentration of 1 mM) is added to the wash buffer strictly before use.

**9. Elution buffer with maltose**

**Table:** 

Reagent	Final concentration	Quantity or Volume
Tris-HCl pH 7.5 (1 M)	20 mM	4 mL
NaCl (4 M)	200 mM	10 mL
Maltose (500 mM)	40 mM	16 mL
dH_2_O	n/a	170 mL
Total		**200 mL**
**Add before use:**		
DTT (1 M)	1 mM	200 μL

Prepare fresh. The prepared solution can be stored at 4 °C and used for 6 months. DTT (to a final concentration of 1 mM) is added to the wash buffer strictly before use.

**10. Maltose-free elution buffer**

**Table:** 

Reagent	Final concentration	Quantity or Volume
Tris-HCl pH 7.5 (1 M)	20 mM	4 mL
NaCl (4 M)	200 mM	10 mL
dH_2_O	n/a	186 mL
Total		**200 mL**
**Add before use:**		
DTT (1 M)	1 mM	200 μL

Prepare fresh. The prepared solution can be stored at 4 °C and used for 6 months. DTT (to a final concentration of 1 mM) is added to the wash buffer strictly before use.

**11. Buffer A**

**Table:** 

Reagent	Final concentration	Quantity or Volume
Tris-HCl pH 7.5 (1 M)	20 mM	4 mL
dH_2_O	n/a	196 mL
Total		**200 mL**
**Add before use:**		
DTT (1 M)	1 mM	200 μL

Prepare fresh. DTT (to a final concentration of 1 mM) is added to buffer A strictly before use.

**12. Buffer B**

**Table:** 

Reagent	Final concentration	Quantity or Volume
Tris-HCl pH 7.5 (1 M)	20 mM	4 mL
NaCl (4 M)	500 mM	25 mL
dH_2_O	n/a	171 mL
Total		**200 mL**
**Add before use:**		
DTT (1 M)	1 mM	200 μL

Prepare fresh. DTT (to a final concentration of 1 mM) is added to buffer B strictly before use.

**13. PIPES 250 mM, pH 7.5**

**Table:** 

Reagent	Final concentration	Quantity or Volume
PIPES	250 mM	3.78 g
Adjust to pH 7.5 with NaOH 5 M		3.5 mL
dH_2_O	to 50 mL	
Total		**50 mL**

Wait until the solution reaches room temperature, then measure the pH. Adjust the pH by adding NaOH 5 M. Begin by adding 3.5 mL and then fine-adjust if needed. Sterilize using a 0.22 μm filter and store at 4 °C.

**14. Tris-HCl, 1 M, pH 7.5**

**Table:** 

Reagent	Final concentration	Quantity or Volume
Tris base	1 M	24.22 g
dH_2_O	n/a	150 mL
Adjust to pH 7.5 with HCl		13 mL
dH_2_O	to 200 mL	
Total		**200 mL**

Wait until the solution reaches room temperature, then measure the pH. Adjust the pH by adding the concentrated HCl. Begin by adding 13 mL and then fine-adjust if needed. Autoclave and store at RT.

**15. Tris-HCl, 1 M, pH 7.4**

**Table:** 

Reagent	Final concentration	Quantity or Volume
Tris base	1 M	24.22 g
dH_2_O	n/a	150 mL
Adjust to pH 7.5 with HCl		15 mL
dH_2_O	to 200 mL	
Total		**200 mL**

Wait until the solution reaches room temperature, then measure the pH. Adjust the pH by adding the concentrated HCl. Begin by adding 15 mL and then fine-adjust if needed. Autoclave and store at RT.

**16. Tris-HCl, 1 M, pH 6.8**

**Table:** 

Reagent	Final concentration	Quantity or Volume
Tris base	1 M	36.33 g
Adjust to pH 7.5 with HCl		24 mL
dH_2_O	to 200 mL	
Total		**200 mL**

Wait until the solution reaches room temperature, then measure the pH. Adjust the pH by adding the concentrated HCl. Begin by adding 24 mL and then fine-adjust if needed. Autoclave and store at RT.

**17. NaCl, 4 M**

**Table:** 

Reagent	Final concentration	Quantity or Volume
NaCl	4 M (0.234 g/mL)	11.69 g
dH_2_O	to 50 mL	
Total		**50 mL**

**18. NaOH, 5 M**

**Table:** 

Reagent	Final concentration	Quantity or Volume
NaOH	5 M (0.2 g/mL)	10 g
dH_2_O	to 50 mL	
Total		**50 mL**

**19. CaCl_2_, 1.5 M**

**Table:** 

Reagent	Final concentration	Quantity or Volume
CaCl_2_·2H_2_O	1.5 M (0.22 g/mL)	11.03 g
dH_2_O	to 50 mL	
Total		**50 mL**

**20. MnCl_2_, 1 M**

**Table:** 

Reagent	Final concentration	Quantity or Volume
MnCl_2_·4H_2_O	1 M (0.2 g/mL)	9.9 g
dH_2_O	to 50 mL	
Total		**50 mL**

**21. KCl, 2.5 M**

**Table:** 

Reagent	Final concentration	Quantity or Volume
KCl	2.5 M (0.19 g/mL)	9.32 g
dH_2_O	to 50 mL	
Total		**50 mL**

**22. MgSO_4_, 1 M**

**Table:** 

Reagent	Final concentration	Quantity or Volume
MgSO_4_·7H_2_O	1 M (0.246 g/mL)	12.3 g
dH_2_O	to 50 mL	
Total		**50 mL**

Sterilize using a 0.22 μm filter. Store at 4 °C.

**23. Maltose, 1 M**

**Table:** 

Reagent	Final concentration	Quantity or Volume
D(+)-Maltose monohydrate	0.5 M (0.18 g/mL)	9 g
dH_2_O	to 50 mL	
Total		**50 mL**

Sterilize using a 0.22 μm filter. Store at 4 °C.

**24. Na_3_C_6_H_5_O_7_, 1 M**

**Table:** 

Reagent	Final concentration	Quantity or Volume
Na_3_C_6_H_5_O_7_·2H_2_O	1 M (0.29 g/mL)	14.7 g
dH_2_O	to 50 mL	
Total		**50 mL**

Store at RT.

**25. IPTG, 1M**

**Table:** 

Reagent	Final concentration	Quantity or Volume
IPTG	1 M (0.24 g/mL)	12 g
dH_2_O	to 50 mL	
Total		**50 mL**

Sterilize using a 0.22 μm filter. Prepare 10 mL aliquots and store at -20 °C. Stable for at least one year.

**26. DTT, 1M**

**Table:** 

Reagent	Final concentration	Quantity or Volume
DTT	1 M (0.154 g/mL)	1.54 g
dH_2_O	to 10 mL	
Total		**10 mL**

Prepare 1 mL aliquots and store at -20 °C. Stable for at least one year.

**27. Ampicillin 1,000×**

**Table:** 

Reagent	Final concentration	Quantity or Volume
Ampicillin	100 mg/mL	5 g
Ethanol	50% (v/v)	25 mL
dH_2_O	n/a	25 mL
Total		**50 mL**

Sterilize using a 0.22 μm filter. Prepare 500 μL aliquots and store at -20 °C. Stable for at least one year.

**28. Glucose, 40% (w/v)**

**Table:** 

Reagent	Final concentration	Quantity or Volume
Glucose	40% (w/v)	20 g
dH_2_O	to 50 mL	
Total		**50 mL**

Autoclave at 121 °C for 15 min. Store at RT.

**29. SDS, 10% (w/v)**

**Table:** 

Reagent	Final concentration	Quantity or Volume
SDS	10% (w/v)	5 g
dH_2_O	to 50 mL	
Total		**50 mL**

Store at RT.

**30. APS, 10% (w/v)**

**Table:** 

Reagent	Final concentration	Quantity or Volume
APS	10% (w/v)	0.2 g
dH_2_O	to 2 mL	
Total		**2 mL**

**31. 4× Loading buffer (SLB)**

**Table:** 

Reagent	Final concentration	Quantity or Volume
SDS (10%)	4% (v/v)	4 mL
Glycerol	20% (v/v)	2 mL
Bromophenol blue	0.004% (w/v)	0.4 mg
Tris-HCl pH 6.8 (1M)	0.125 M	1.25 mL
β-mercaptoethanol**	5%	0.5 mL
dH_2_O	to 10 mL	2.75 mL
Total		**10 mL**

Store at -20 °C for up to one year. Before use, warm the buffer slightly until any SDS crystals disappear. Add 100 μL of β-mercaptoethanol to 900 μL of 4× SLB and mix well.

**32. 1× Electrode buffer (Tris-Glycine/SDS) pH 8.3**

**Table:** 

Reagent	Final concentration	Quantity or Volume
Tris-base	25 mM	3.03 g
Glycine	190 mM	14.4 g
SDS	0.1% (w/v)	1 g
dH_2_O	n/a	to 1 L
Total		**1 L**

**33. Acrylamide/Bis-acrylamide, 30% solution**

**Table:** 

Reagent	Final concentration	Quantity or Volume
Acrylamide	29% (w/v)	14.5 g
Bis-acrylamide	1% (w/v)	0.5 g
dH_2_O	to 50 mL	
Total		**50 mL**

Sterilize using a 0.45 μm filter.

**34. Resolving gel buffer pH 8.8**

**Table:** 

Reagent	Final concentration	Quantity or Volume
Tris-base	1.5 M	36.3 g
Adjust to pH 8.8 with HCl*		10.5 mL
dH_2_O	n/a	to 200 mL
Total		**200 mL**

Wait until the solution reaches room temperature, then measure the pH. Store at RT.

**35. Stacking gel buffer pH 6.8**

**Table:** 

Reagent	Final concentration	Quantity or Volume
Tris-base	0.5 M	3.025 g
Adjust to pH 6.8 with HCl*		2.1 mL
dH_2_O	n/a	to 50 mL
Total		**50 mL**

Wait until the solution reaches room temperature, then measure the pH. Store at RT.

**36. 20% PEG 8000**

**Table:** 

Reagent	Final concentration	Quantity or Volume
PEG 8000	20% (w/v)	0.4 g
Tris-HCl, 20 mM, pH 7.4	to 2 mL	
Total		**2 mL**

Store at -20 °C for up to 2 months.

**Laboratory supplies**

1. SnakeSkin^TM^ dialysis tubing, 16 mm, 10 kDa (Thermo Scientific, catalog number: 26616)

2. Affinity chromatography column 12 mL (MedChemExpress, catalog number: HY-K0221)

3. 50 mL high-speed conical centrifuge tubes (Corning-Costar, catalog number: 430829)

4. Centrifuge bottle assembly, polypropylene 500 mL (Beckman, catalog number: 355607)

5. Thermo Scientific Nalgene Oak Ridge high-speed PPCO centrifuge tubes 50 mL (Thermo Fisher Scientific, catalog number: 10757411)

6. Microtubes for Qubit^TM^ 4 fluorometer, 5 mL (Thermo Fisher Scientific, catalog number: Q32856)

7. 0.22 μm syringe filters (Corning-Costar, catalog number: 431229)

8. 0.45 μm syringe filters (Corning-Costar, catalog number: 431220)

9. Amicon Ultra concentrators-15 10K (Merсk Millipore, catalog number: UFC901024)

10. Microscope slides super grade (Citotest, catalog number: 0303)

11. Cover glasses 15 × 15 mm (Menzel-glaser, catalog number: 10.0360.82)

12. Ice

13. Standard molecular biology consumables (tubes, tips, serological pipettes, Petri dish, Parafilm, scissors, tweezers, sterile loop, syringe, sterile flasks 2 L and 250 mL)

## Equipment

1. 44R incubator shaker (New Brunswick, Innova, catalog number: M1282-0006)

2. Centrifuge 5804 R (Eppendorf, catalog number: 5805000620)

3. Centrifuge Sorvall LYNX 4000 (Thermo Fisher Scientific)

4. Doc-Print VX5 (Vilber Lourmat)

5. iBright CL1500 Imaging System (Thermo Fisher Scientific)

6. Laminar hood for bacterial cultivation

7. Magnetic stirrer Heidolph (Heidolph, catalog number: 505-20000-00-5)

8. pH meter PP-15 (Sartorius)

9. Pipet filler S1 (Thermo Fisher Scientific, catalog number: 9501)

10. Pipetman P20, P200, P1000 (Gilson, catalog numbers: F123600, F123601, F123602)

11. PowerPac basic power supply (Bio-Rad, catalog number: 1645050EDU)

12. Qubit^TM^ 4 fluorometer (Thermo Fisher Scientific, model: Q33226)

13. SDS-PAGE equipment, Mini-PROTEAN Tetra cell (Bio-Rad, catalog number: 1658001)

14. Shaker Heidolph unimax 1010 (Heidolph, catalog number: 543-12310-00-4)

15. UV transilluminator (Vilber Lourmat)

16. Tube Rotator Intelli-Mixer RМ (Elmi)

17. VirSonic100 (ultrasonic cell disrupter) (VirTis, catalog number: 217640)

18. Water bath

19. Zeiss AxioScope A.1 fluorescence microscope (Zeiss)

20. Bacteria incubator at 37 °C

21. Akta Pure chromatography system (GE Healthcare)

## Software and datasets

1. ImageJ/Fiji (Free, NIH)

2. ZEN 3.2 Blue edition (Zeiss)

3. Snapgene (GSL biotech LLC, CA, USA)

## Procedure

**A. Verification of peptide expression (takes 4 days)**

The plasmid pMAL_Treacle 2**×** LCR (obtained in [7]) was used for the expression of a 136 amino acid peptide corresponding to a fragment of the treacle protein (residues 291–426). This region includes two serine/glutamate-rich low-complexity regions (LCRs) and two intervening linker sequences. The expression vector p-MAL encodes MBP at the N-terminus, followed by a TEV protease cleavage site and a treacle DNA fragment at the C-terminus. MBP increases protein solubility and is not oligomerized, unlike GST. At the initial stage, it is necessary to check the expression of the peptide in small volumes using the pull-down method.

**1. Transformation of BL21 (DE3) with the expression vector (takes 1 day)**

**Day 1**

*Note: Work in a safety cabinet.*

a. Prepare a fresh stock culture of bacterial strain BL21 (DE3). BL21 should be grown on an LB plate without antibiotics, and a fresh transfer should be made once a month.

*Note: BL21 cells must be fresh, since cells from old stock cultures do not grow or transform poorly.*

b. Transfer BL21 from a fresh stock culture into 1 mL of Terrific Broth medium using a sterile loop. Incubate the culture at 37 °C with shaking at 600× *g* for 1–1.5 h until OD600 = 0.1–0.3.

*Note: Optical density (OD) is measured using a spectrophotometer relative to Terrific Broth medium without BL21, wavelength 600 nm*.

c. Pellet the cells at 2,300× *g* for 5 min at 4 °C and transfer the samples to ice.

*Note: All subsequent manipulations with cells are carried out on ice.*

d. Gently resuspend the pellet in 1 mL of TB buffer and incubate on ice for 10 min.

e. Pellet the cells at 6,000× *g* for 30 s at 4 °C.

f. Gently resuspend the pellet in 70 μL of TB buffer and very carefully/gently add 1 µg of plasmid pMAL_Treacle 2× LCR. Incubate on ice for 30 min.

g. Transfer samples to a 42 °C water bath for 2 min and immediately transfer them to ice. Incubate on ice for 1 min.

h. Optional. Add 1 mL of Terrific Broth medium to the samples and incubate at 37 °C with shaking at 600× rpm for 1 h. *Note: This is particularly important when working with plasmids carrying genes for resistance to translation-inhibiting antibiotics (e.g., kanamycin, streptomycin, and chloramphenicol), as these antibiotics act by blocking protein synthesis, which can slow cell growth and reduce the number of transformed cells. In contrast, for ampicillin resistance, this additional growth step is usually not required.*

i. Transfer samples to LB plates with 0.5% glucose containing 100 μg/mL ampicillin and spread. Optional: Apply 10% and 90% of the cell suspension to dishes to select the optimal density of bacterial colony growth.

*Note: Glucose suppresses background transcription from the promoter.*

**2. Protein extraction in small volumes using the pull-down method (takes 2 days)**

**Day 2**

*Note: Work in a safety cabinet.*

a. Resuspend 10–20 transformed bacterial colonies from LB plates in 1 mL of Terrific Broth medium and transfer to 50 mL of Terrific Broth medium containing 100 µg/mL ampicillin. Grow at 37 °C with shaking at 200× *g* until OD600 = 1–1.5.

*Note: Use sterile flasks with a volume of 250 mL.*

b. Cool samples to RT. Take aliquot #0 of 250 µL of bacterial culture (before IPTG induction). Pellet the aliquot at 5,000× *g* for 5 min at 4 °C and resuspend in 25 µL of start buffer. Aliquot #0 should be stored at -20 °C.

c. To the sample, add 50 μL of 1 M IPTG (1 mM final concentration) and incubate overnight at 18 °C with shaking at 250× *g*.

*Note: IPTG induces the expression of phage T7 RNA Polymerase, which in turn binds to the T7 promoter in plasmids, providing highly efficient expression of the target protein in BL21(DE3) bacteria.*

**Day 3**

*Note: Work on a lab bench.*

d. Pellet the cells at 3,200× *g* for 20 min at 4 °C.

e. Gently resuspend the pellet in 2 mL of start buffer. Take aliquot #1 of 25 µL of bacterial culture (after IPTG induction). Aliquot #1 should be stored at -20 °C.

f. Disrupt the cells by sonication in start buffer on ice. Pre-cool the sample on ice for 5 min, then sonicate using 5 cycles of 20 s on and 2 min off at 50% amplitude.

*Note: Pre-cooling of samples prevents overheating of samples during sonication. Avoid foaming the sample. The crushing mode is optimally selected for different models of sonicators.*

g. Pellet the lysate at 16,000× *g* for 20 min at 4 °C. Collect the supernatant and transfer to ice.

h. Using a carefully cut nozzle tip, take 50 μL of the amylose resin and transfer it to a tube with 1 mL of start buffer. Incubate for 5 min at 4 °C to equilibrate buffer conditions.

*Note: The amylose resin solution is thick. The solution must be collected with tips whose inlet is slightly shaped by scissors or tips with a wide opening.*

i. Pellet the amylose resin at 1,000× *g* for 1 min at 4 °C. Remove supernatant.

j. Transfer lysate-supernatant to the amylose resin and incubate with smooth mixing on the rotor at 20× *g* for 10 min at 4 °C.

*Note: Incubation time can be increased to 1 h. It is convenient to use the rotor tube rotator Intelli-Mixer RМ.*

k. Pellet the amylose resin at 1000× g for 1 min at 4 °C. Remove supernatant.

l. Wash the amylose resin three times by the addition of 1 mL of wash buffer and centrifuge at 1,000× *g* for 1 min at 4 °C.

m. Elute peptides by adding 150 μL of elution buffer and 50 μL of 4× SLB to the resin. Mix and boil the sample for 5 min.

n. Pellet the amylose resin at 1,000× *g* for 1 min at 4 °C. Transfer the supernatant to a new collection tube.

o. Take aliquot #2 of 25 μL elution sample. Aliquot #2 should be stored at -20 °C.

p. Thaw aliquots #0 and #1 at RT and added 9 μL of 4× SLB. Mix and boil samples for 5 min. Aliquots should be stored at -20 °C.

**3. Analysis of protein expression by gel electrophoresis followed by staining (takes 1 day)**

**Day 4**

*Note: Work on a lab bench. It is convenient to use SDS-PAGE equipment and Mini-PROTEAN Tetra cell.*

a. Prepare 10% resolving and 4% stacking SDS-PAGE gel according to the manufacturer's recommendations of Abcam or Bio-Rad. Use 1× electrode buffer. Place the gel in a chamber.

b. Add 4**×** SLB buffer to each sample and incubate in a boiling water bath for 5 min.

c. Apply samples in wells. Aliquot #0 (before IPTG induction) at 5 μL, aliquot #1 (after IPTG induction) at 5 μL, aliquot #2 (after amylose resin) at 20 μL, and protein ladder.

d. Perform electrophoresis at 20 mA on the gel for 30 min at RT and then at 40 mA on the gel until the samples reach the lower boundary of the gel.

e. Transfer the gel into 100 mL of protein staining solution (One-Step Blue Protein Gel Stain). Incubate for 1 h at RT with gentle stirring at 50× *g* on a shaker or overnight at 4 °C.

*Note: The Coomassie Brilliant Blue G-250 dye, the core of the assay, binds to basic amino acids (lysine, arginine, and histidine), particularly in an acidic environment, leading to a color change that correlates with protein concentration. This specificity means the assay's accuracy can be affected if a protein has an unusual abundance or scarcity of these basic residues. Since treacle peptide contains 12.5% essential amino acids, it is preferable to stain it in gel using a One-Step Blue Protein Gel Stain. The One-Step Blue Protein Gel Stain gives a brighter color to the gel than the Coomassie Brilliant Blue G-250 dye.*

**Figure 2.**
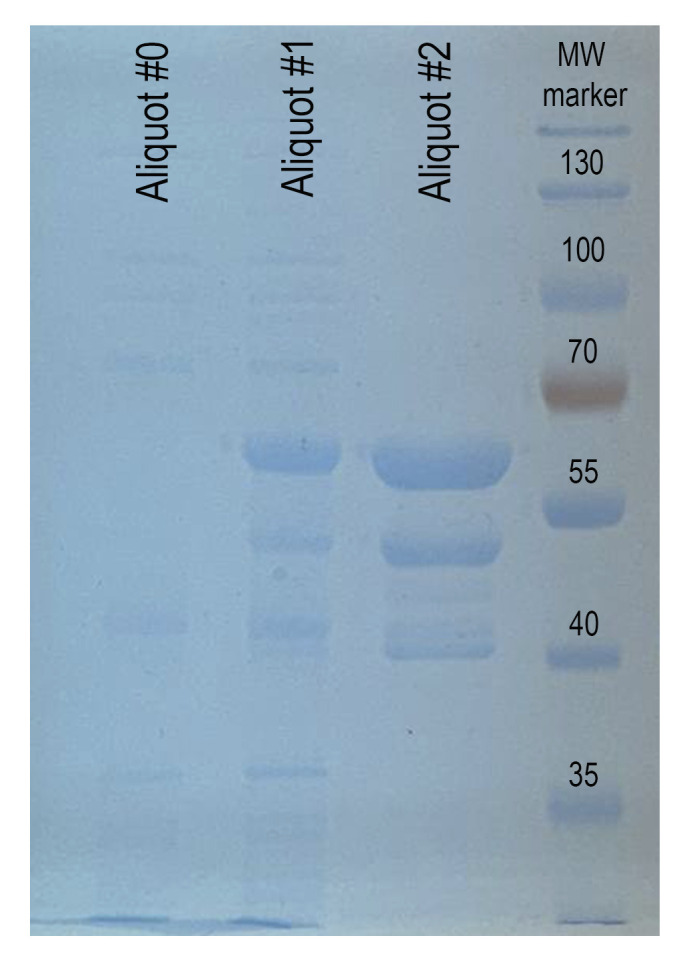
Expression and affinity purification of the recombinant MBP-treacle fragment. SDS-PAGE analysis of the expression and purification of the MBP-treacle fusion protein (uncut fragment), visualized using One-Step Blue protein stain. Aliquot #0: Total bacterial lysate from *E. coli* culture without IPTG induction (negative control). Aliquot #1: Total lysate following IPTG induction, showing a prominent band corresponding to the overexpressed recombinant MBP-treacle fragment. Aliquot #2: Eluate obtained after affinity purification using amylose resin, demonstrating enrichment of the target fusion protein. MW marker: Protein molecular weight marker.

f. Rinse the gel twice in 50 mL of water. Move the membrane to the transilluminator and take a photo of the gel. Expect prominent bands of MBP-Treacle 2**×**LCR peptide at 65 kDa after IPTG induction (Figure 2).

*Note: MBP-Treacle 2× LCR peptide 65 kDa is a fusion peptide of treacle and MBP with an unclipped MBP domain.*

**B. Protein fragment purification (takes 7 days)**

Once you are sure that the peptide is produced in sufficient quantities, proceed with peptide purification. The first stage involves producing large volumes of bacterial lysate. This is followed by a resin purification stage and cutting off the MBP from the target peptide. The final stage involves dialyzing and further purifying the peptide on a chromatography column.

**1. Peptide expression in large quantities (takes 2 days)**

**Day 1**

*Note: Work in a safety cabinet.*

a. Prepare 1 L of Terrific Broth medium. Pour 1 L of the medium into two sterile 2 L flasks (500 mL each).

*Note: Two flasks are used to ensure equivalent equilibrium in subsequent procedures. For one sample extraction, 500 mL of medium is sufficient. 500 mL is used for peptide production. The second Falcon can be poured out or used as a spare.*

b. Resuspend 20–50 transformed bacterial colonies from LB plates in 1 mL of Terrific Broth medium. Transfer 500 μL of bacterial suspension to 500 mL of Terrific Broth medium containing 100 µg/mL ampicillin. Grow at 37 °C with shaking at 220× *g* until OD600 = 1–1.5.

c. Cool samples to RT. Take an aliquot of 250 μL of bacterial culture (before IPTG induction) for induction control as described in step A2b.

d. To the sample, add 500 μL of 1 M IPTG (1 mM final concentration) and incubate overnight at 18 °C with shaking at 220× *g*.

*Note: Make sure the flasks are securely fastened*.

**Day 2**

*Note: Work on a lab bench.*

e. Take an aliquot of 250 μL of bacterial culture (after IPTG induction) for induction control as described in step A2e.

f. Pour no more than 300 mL of bacterial suspension into centrifuge cups (500 mL volume) and spin at 5,000× *g* for 20 min at 4 °C. Drain the supernatant and add the remaining volume of bacterial suspension to the same cups. Repeat centrifugation.

*Note: If necessary, the dry sediment can be frozen at -80 °C in 50 mL Falcon tubes using liquid nitrogen.*

g. Resuspend the cell pellet in 10 mL of ice-cold start buffer containing inhibitors (at a rate of 500 mL of bacterial suspension per 10 mL of start buffer). Transfer to 50 mL Falcon tubes.

h. Incubate on ice for 5 min.

i. Disrupt the cells by sonication in start buffer on ice. Pre-cool the sample on ice for 5 min, then sonicate using 20 s on and 2 min off at 50% amplitude, and then 5 cycles for 20 s on and 2 min off at 100% amplitude.

*Note: Pre-cooling of samples prevents overheating during sonication. Avoid foaming the sample.*

j. Pellet the lysate at 20,000× *g* for 60 min at 4 °C. Collect the supernatant and transfer on ice.

**2. Affinity purification and concentration (takes 3 days)**

**Day 3**

*Note: Work on a lab bench.*

a. Prepare the amylose resin gravity flow column according to the manufacturer’s instructions.

*Note: Subsequent procedures on the column should be carried out in a refrigerator at 4 °C.*

b. Equilibrate the amylose resin gravity flow column with 10 column volumes of start buffer. Wait until the buffer has passed through the column.

c. Apply the supernatant from step B1j to the column. Wait until the supernatant has passed through the column.

d. Wash the column with more than 10 column volumes of wash buffer. Wait until the wash buffer has passed through the column.

e. Cap the column. Apply 5 mL of maltose-free elution buffer containing 5 mM sodium citrate and TEV-protease according to the manufacturer's recommendations.

*Note:1 unit of TEV Protease will cleave 2 µg of MBP-fusion protein. The calculated amount of protease is 1,000 U (100 μL). It is possible to add more protease, since the activity of the ferment may decrease with storage time.*

f. Place the sealed column in a 50 mL Falcon tube and incubate overnight on a rotator at 20× *g* at 4 °C.

**Day 4**

g. Collect the fraction from the column and add another 5 mL of maltose-free elution buffer. Combine the fractions and transfer to ice. The target peptide is enriched in this fraction, although some MBP is still present as an impurity.

h. Elute MBP from the column by the addition of 10 mL of elution buffer with maltose.

i. Transfer 10 mL of the peptide fraction to the dialysis tubing for a 10 kDa molecular weight cutoff (MWCO). Dialyze against 1 L of 20 mM Tris-HCl, pH 7.4, overnight on a magnetic stirrer at 150× *g* at 4 °C.

*Note: The pore size is selected based on the protein molecular weight to prevent leakage during dialysis. A membrane with a cutoff below 10 kDa is typically suitable.*

**Day 5**

j. Transfer the contents of the dialysis bag to 50 mL Falcon tubes and centrifuge at 13,000× *g* for 10 min at 4 °C. Take aliquot #0 (Input) of 20 μL of peptide fraction.

*Note: This stage is necessary to purify the solution from aggregates of the peptide.*

**3. Ion-exchange chromatography and concentration (takes 2 days)**

**Day 6**

*Note: Work on a lab bench.*

**Figure 3.**
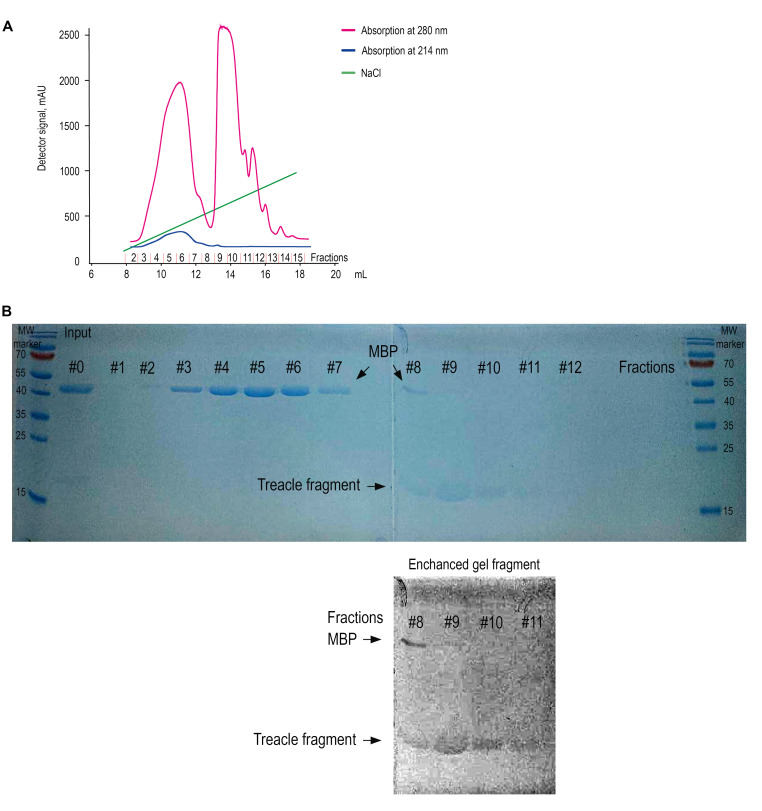
Ion-exchange purification and detection of the recombinant treacle fragment by One-Step Blue staining. (A) Ion-exchange chromatogram. The chromatographic profile displays two distinct absorbance wavelengths: 280 nm (pink trace, primarily monitoring aromatic amino acids) and 214 nm (blue trace, detecting peptide bond absorption). Quantitative analysis was performed using the 214 nm signal due to its superior sensitivity for peptide detection. The green line indicates the increasing concentration of NaCl. Elution of the target treacle peptide commenced at 265 mM NaCl, as evidenced by the characteristic rise in absorbance at this ionic strength. The numbers of the collected fractions on the X-axis correspond to the numbers of the fractions on the SDS-PAGE analysis. (B) SDS-PAGE analysis of fractions collected during ion-exchange chromatography, visualized using One-Step Blue Protein Gel Stain. The input sample (lane *Input*) contains a mixture of the recombinant treacle fragment and cleaved MBP (maltose-binding protein). Lanes 1–12 correspond to sequential fractions eluted with a NaCl gradient. MBP (~42.5 kDa) is predominantly found in early fractions (lanes 1–7), whereas the treacle fragment, which migrates slightly above the 15 kDa marker, is enriched in fractions 9–11. Due to its low aromatic amino acid content, the treacle fragment stains weakly. To aid detection, the bottom panel shows a digitally enhanced section of the gel, revealing the position of the treacle fragment. Fraction 9, containing the highest enrichment of the target peptide, was selected for dialysis and further use. MW marker: Protein molecular weight marker.

a. Equilibrate MonoQ 5/50 GL (Cytiva) with 5–10 column volumes of buffer A.

b. Apply the dialyzed protein from step B2j to the MonoQ 5/50 GL (Cytiva). Collect the flowthrough.

c. Wash the column with more than 10 column volumes of buffer A.

d. Elute the bound protein using a 50–500 mM linear NaCl gradient over 30 column volumes at a flow rate of 0.5–1 mL/min (Figure 3A). Collect 0.75 mL fractions (#1–12) and analyze by gel electrophoresis (Figure 3B).

e. Prepare 15% resolving and 4% stacking SDS-PAGE gel and perform electrophoresis as described in step A3a (Figure 3B).

f. Dialyze eluted proteins (fraction #9) at 4 °C against assay buffer (20 mM Tris-HCl pH 7.4) at 4 °C overnight on a magnetic stirrer at 150× *g.*

*Note: The expected yield is 500–1,000 mg of protein per liter. The volume for dialysis is 3–4 mL.*

**Day 7**

g. Centrifuge peptide solution at 13,000× *g* for 10 min at 4 °C.

*Note: This stage is necessary to purify the solution from aggregates of the peptide.*

h. Prepare Amicon Ultra concentrators according to the manufacturer's recommendations.

i. Transfer the sample to a 50 mL Amicon filter with a pore diameter of 10 K. Centrifuge at 5,000× *g* for 10–20 min at 4 °C until the volume of liquid in the filter reaches 1 mL.

j. Measure the protein concentration (mg/L) on the Qubit^TM^ fluorometer using the Qubit^TM^ Protein BR Assay kit according to the manufacturer’s instructions.

*Note: The Bradford assay's sensitivity to specific amino acids lies in its interaction with basic amino acids: lysine (K), arginine (R), and histidine (H). Since treacle peptide contains only 12.5% basic amino acids, the Bradford assay gives false results. For measuring peptide concentration, it is preferable to use a Q-beat. The expected protein yield ranges from 1,000 to 3,000 mg per liter of 500 mL culture. This would typically yield 1 mL of concentrated protein solution.*

k. Freeze aliquots in liquid nitrogen and store peptide solution at -80 °C. The purified fragment remains stable at -80 °C for extended periods. Repeated freeze-thaw cycles are not recommended.

**C. In vitro treacle fragment condensation analysis (takes 1 day)**

*Note: Work on a lab bench.*

**Figure 4.**
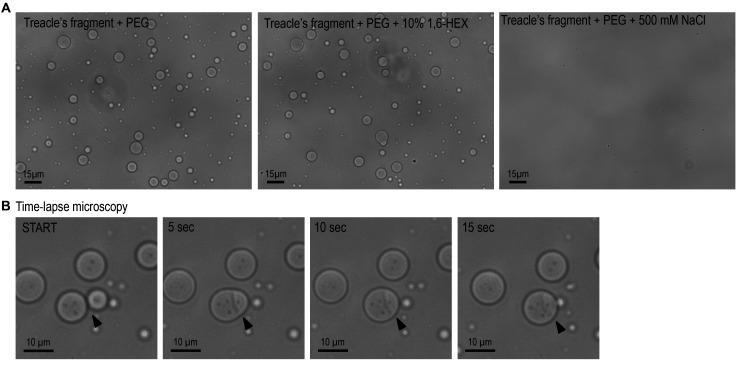
In vitro phase separation and fusion dynamics of a recombinant treacle fragment. (A) The purified recombinant treacle fragment forms condensates in vitro in the presence of 5% polyethylene glycol (PEG). Phase separation is inhibited by 500 mM NaCl, suggesting a predominant role of electrostatic interactions. No significant effect is observed upon treatment with 10% 1,6-hexanediol (1,6-HEX), indicating limited contribution of hydrophobic interactions. (B) Time-lapse microscopy captures fusion events between individual treacle condensates, consistent with their liquid-like behavior and dynamic rearrangement over time. Arrows mark the sites of condensate coalescence.

a. Dilute treacle peptide in 20 mM Tris-HCl pH 7.4 to a final concentration of 0.6–1 mg/mL.

b. To assemble condensates, add PEG 8000 to the protein solution to a final concentration of 5%, followed by incubation at RT for 10 min.

c. Pipette 10 μL of the peptide solution onto a glass slide, cover with a coverslip, and analyze on the Zeiss AxioScope A.1 fluorescence microscope in DIC mode I (Figure 4).

d. To test the effects of NaCl or 1,6-hexandiol on treacle condensation, add reagents to the protein solution with 5% PEG 8000 at 500 mM NaCl or 10% 1,6-hexandiol concentrations (Figure 4).

## Data analysis

Most microscope imaging software enables the placement of a scale bar on micrographs, which can subsequently be used for spatial calibration. Following calibration, the size of multiple condensates can be quantitatively analyzed using ImageJ. By acquiring time-lapse images at intervals of 1–2 s, the dynamic fusion of individual condensates into larger assemblies can be observed. Sequential frames can then be compiled into a time-lapse video using ImageJ to visualize the kinetics of condensate coalescence.

## General notes and troubleshooting

The efficiency of recombinant protein expression and purification is highly dependent on the intrinsic physicochemical properties of the target protein. Consequently, empirical optimization of experimental parameters is required for each individual case [8]. In this section, we systematically outline prevalent challenges in heterologous protein production and purification workflows, accompanied by evidence-based troubleshooting strategies.

**Troubleshooting**

**Table:** 

Low yield	Optimize expression conditions. Test lower induction temperatures (e.g., 16–25 °C); lower temperatures can enhance solubility and minimize inclusion body formation. Optimize IPTG concentration, as both under- and over-induction can reduce protein yield. Use codon-optimized constructs. Ensure the gene sequence is optimized for bacterial codon usage. Try different strains, such as Rosetta. For optimal peptide binding to the ion-exchange resin, apply the sample at a minimum volume of 10 mL. Reduced loading volumes adversely affect both the purity and recovery yield of the eluted product.
Poor binding	Optimize binding conditions. Adjust pH and salt concentration in the start buffer to ensure they fall within the optimal range for your resin. Pre-clear the lysate by centrifugation/filtration to reduce clogging. Increase binding capacity: Reduce flow rate during loading (e.g., 0.5–1 mL/min) or use a larger column volume if the protein exceeds the resin’s binding capacity. Evaluate resin/column issues: Regenerate the resin if reused, or switch to a different type. Ensure no air bubbles or channeling disrupt the flow.
No TEV protease cleavage	Optimize cleavage conditions. Extend incubation time (e.g., overnight at 4 °C), adjust the protease:substrate ratio (typically 1:20–1:50), or include reducing agents (e.g., 1–5 mM DTT) to maintain TEV activity. Verify protease quality. Use fresh, active TEV protease (check by SDS-PAGE/Coomassie) or switch to a more efficient variant (e.g., His-tagged TurboTEV).
No LLPS	It is necessary to check the purity and quality of the isolated peptide. The concentration of the peptide should be high enough, ideally more than 600 mg/L. The buffer in which the protein is dissolved should not contain salt and foreign impurities. To demonstrate LLPS, do not forget to add the crowding agent 5% dextran sulfate.
No visible protein on gel	When staining a protein in a gel, the peptide is weakly detected. This is a property of this peptide, directly dependent on the amino acid composition. The most suitable methods for staining the peptide are One-Step Blue Protein Gel Stain overnight or standard silver staining.

## Validation of protocol

This protocol (or parts of it) has been used and validated in the following research article:

- Velichko et al. [7] Treacle's ability to form liquid-like phase condensates is essential for nucleolar fibrillar center assembly, efficient rRNA transcription and processing, and rRNA gene repair. *eLife* (Figure 2B,C; Figure 4, Figure supplement 1B).
